# Editorial: Autonomic nervous system and cardiovascular risk

**DOI:** 10.3389/fnins.2023.1185320

**Published:** 2023-04-04

**Authors:** Vitor E. Valenti, Luiz C. M. Vanderlei

**Affiliations:** ^1^Autonomic Nervous System Center, São Paulo State University (UNESP), Marilia, SP, Brazil; ^2^Department of Physical Therapy, Faculty of Sciences and Technologies, São Paulo State University (UNESP), Presidente Prudente, SP, Brazil

**Keywords:** autonomic nervous system, cardiovascular, neuroscience, cardiovascular risk, sympathetic, vagus, parasympathetic tone

The autonomic nervous system is related to involuntary body functions, including gastrointestinal mechanisms, blood pressure, skin muscle activity and heart rate (Hall, 2015). The literature provided strong evidence that autonomic dysfunction may lead to cardiovascular risk factors (Gezer et al., 2022; Samora et al., 2023). Accordingly, this Research Topic explores recent studies that investigated the relationship between the autonomic nervous system and cardiovascular risk.

The first article published in this Research Topic was the study conducted by Abdalbari et al., which applied Granger Causality to capture brain-heart mechanisms during wakefulness and sleep. Granger Causality is a statistical test that can also be used as a measure for bidirectional connectivity in EEG (Coben and Mohammad-Rezazadeh, 2015). In the Abdalbari et al. study, EEG and ECG were recorded during the different sleep stages and wakefulness. As the main data, the authors demonstrated the relevance of fronto-posterior connectivity during wakefulness and sleep stages *via* Granger Causality. The study also showed differences in ipsilateral and contralateral mechanisms and identified bidirectional brain-heart connections which are stronger from brain to heart.

Brain-heart interaction was also evaluated by Liu Y. et al.. These authors analyzed alterations in spontaneous function of the brain, which are related to autonomic function, during changes in the cardiovascular system induced by dobutamine, a sympathomimetic drug usually used for heart failure treatment (alpha-1, beta-1, and beta-2 agonist drug) (Drugs Lactation Database., 2006; Mahoney et al., 2016). In healthy young subjects cognitive function was unaffected when blood pressure and heart rate increased following dobutamine administration. However, spontaneous neural activity was changed after dobutamine infusion. In addition, the level of spontaneous brain activity was associated with systolic and diastolic arterial pressure changes.

In this context, the vasovagal syncope (VVS), also known as neurocardiogenic syncope or reflex syncope, is an impairment investigated worldwide (Ali et al., 2021). Li et al., provided interesting data regarding the Calcitonin gene-related peptide (CGRP) and its association with VVS. The CGRP is a peptide localized to C and Aδ sensory fibers with important role in sensory and efferent function, which presents a broad innervation throughout the body with large perivascular location (Russell et al., 2014). Li et al. compared the CGRP plasma levels between healthy children and children with VVS. Their data indicated that plasma CGRP concentration was significantly increased in VVS children. Furthermore, correlation analysis evidenced positive association between CGRP and clinical symptoms severity, indicating that CGRP levels estimated treatment efficacy.

Regarding the vagus nerve, Cai et al. performed an overview related to symptomatic vagal-induced sinus node dysfunction. The study described many factors in order to identify the best candidate for cardioneuroablation, a therapeutic option in sinus dysfunction, neurocardiogenic syncope and functional atrio-ventricular block (Aksu et al., 2019). The researchers highlighted cardiac autonomic nervous system mechanisms, sinus node dysfunction with vagal overactivity and cardioneuroablation considered for a new treatment for refractory symptomatic sinus node dysfunction. On the other hand, the review reinforced that most of the studies presented low quality of evidence.

A systematic review was conducted by Aftyka et al. to discuss the estimative power of heart rate variability for stroke course, functional outcome, and medical complications. In this context, heart rate variability is a non-invasive method that estimates heart rhythm autonomic regulation. Increased heart rate variability is related to good adaptation, i.e., healthy individuals with efficient autonomic mechanisms whereas reduced heart rate variability is an indicator of impaired adaptation of the autonomic nervous system (Task Force., 1996). The initial search found 1,305 potential references. After rigorous exclusion criteria based on the PRISMA (Preferred Reporting Items for Systematic Reviews and Meta-Analyses) method, 36 studies that investigated linear and non-linear heart rate variability were included. Following a careful quality assessment and risk of bias evaluation, the systematic review concluded that heart rate variability may be considered an optimal predictor of stroke outcomes and complications. Nevertheless, the authors pointed to individual methodological features to adequately measure and interpret the heart rate variability indices.

Another study related to heart rate variability was published by Liu J. et al. in this Research Topic. The study examined the effect of taxane combined with carboplatin, antineoplasic and cardiotoxic drugs (Griesinger et al., 2019; Guijosa et al., 2022) on heart rate variability in patients with cervical cancer. The patients were divided into one group treated with docetaxel + carboplatin whereas another group received paclitaxel + carboplatin. Short heart rate variability was analyzed *via* standard deviation of normal-to-normal intervals (SDNN), root mean square of successive differences (RMSSD), low-frequency power (LF), high-frequency power (HF), and LF/HF. As the main data, heart rate variability was increased by taxane and carboplatin treatment.

Finally, Takotsubo cardiomyopathy is another pathology involved with the relationship between autonomic injury and cardiovascular risk increase (Amin et al., 2020). With this in mind, Arai et al. tried to establish a new animal model related to stress cardiomyopathy. The study investigated epilepsy-induced stress cardiomyopathy and the involvement of neuropeptide Y in cardiac dysfunction. The report evidenced that activation of the sympathetic nervous system leads to upregulation of neuropeptide Y in cardiac sympathetic nerves and stellate ganglion.

Briefly, the articles published in this Research Topic provide additional information regarding brain-heart interaction, vagus nerve activity, heart rate variability and stress cardiomyopathy. Therefore, the results obtained in this Research Topic supply compelling evidence that adds valid data to better comprehend the association between cardiovascular risk and autonomic nervous system.

## Author contributions

VV and LV drafted the editorial and gave final approval.
